# Cutting balloon fenestration for wire-induced distal vessel dissection: a case report and clinical insights

**DOI:** 10.1097/RC9.0000000000000073

**Published:** 2026-01-20

**Authors:** Jamil Nasrallah, Syed Muhammad Hassan Murshid, Rima Chaddad, Eran Sim, Bharat V Khialani

**Affiliations:** a Department of Medicine, Faculty of Medicine, Lebanese University, Beirut, Lebanon; b Department of Life Sciences, Faculty of Sciences, Lebanese University, Beirut, Lebanon; c Department of Medicine, Abbasi Shaheed Hospital, Karachi, Pakistan; d Department of Cardiology, Grand Hospital de l’Est Francilien, France; e Department of Cardiovascular Medicine, Tan Tock Seng Hospital, National Healthcare Group, Singapore

**Keywords:** coronary artery dissection, cutting balloon, IMH, IVUS, PCI

## Abstract

**Introduction and importance:**

Wire-induced coronary artery injury may result in significant vessel dissection, intramural hematoma (IMH), and loss of distal coronary flow. Urgent intervention may be required to restore coronary perfusion, although managing these cases can be challenging due to technical and procedural limitations.

**Case presentation:**

An 80-year-old male presented with an ST-elevation myocardial infarction and underwent left circumflex artery percutaneous coronary intervention complicated by wire-induced distal dissection, IMH, and VF. Balloon dilatation failed, but intravascular ultrasound (IVUS) guided balloon fenestrations restored the flow, followed by successful stenting.

**Clinical discussion:**

Wire-induced distal coronary dissections are rare but can lead to critical ischemia. IVUS plays a key role in confirming wire position, assessing the extent of hematoma, and guiding precise intervention. Although cutting balloon (CB) fenestration has primarily been described in spontaneous coronary artery dissection, this technique can serve as a valuable bailout strategy in iatrogenic dissections when conventional balloon dilatation fails. Controlled micro-incisions created by the CB enable hematoma drainage and flow restoration while minimizing further vessel trauma, even in small distal segments.

**Conclusion:**

Cutting balloon fenestration under IVUS guidance may represent a feasible bailout strategy for flow-limiting, wire-induced dissections complicated by IMH.

## Introduction

Coronary artery dissections occur when the intimal layer of the vessel is torn, allowing blood to seep into the subintimal space and form an intramural hematoma (IMH). For dissections or hematomas that do not significantly obstruct blood flow, conservative treatment is usually favored. However, when there are flow-limiting dissections, urgent intervention is usually necessary to restore distal flow. A potential approach for managing coronary dissections and IMHs involves using cutting balloons (CB)^[1]^. Most instances documented in the literature involve treating spontaneous coronary artery dissection (SCAD)^[2,3]^. The cutting balloons used were typically either smaller than the vessel’s reference size or matched 1:1 with the vessel diameter and inflated at low or nominal pressures^[4]^. Data on the use of CB fenestration in *iatrogenic*, wire-induced distal vessel dissections are sparse. While cutting balloon fenestration has been described primarily in SCAD, its application as a bailout strategy for iatrogenic, wire-induced distal vessel dissection remains rarely reported. This case extends prior observations by demonstrating the successful use of this technique in an iatrogenic setting. Here, we describe a case demonstrating the utility of such techniques in iatrogenic, wire-induced distal dissections as a bailout to restore vessel flow, after which the vessel can be secured with a stent/scaffold. This manuscript was prepared following the SCARE guidelines^[5]^.

**HIGHLIGHTS**

- Cutting balloon (CB) fenestration restored flow in wire-induced coronary dissection.
- Intravascular ultrasound confirmed lumen position, hematoma drainage, and optimal stent expansion.
- Low-pressure CB inflation minimized vessel trauma in distal small-caliber vessels.
- Technique offers a rescue option when conventional balloon angioplasty fails.

## Case presentation

### Background

An 80-year-old male presented with 1–2 days of chest pain and electrocardiographic evidence of ST-segment elevation, consistent with an ST-elevation myocardial infarction (STEMI). High-sensitivity troponin I levels were elevated. The patient had ongoing chest pain, prompting urgent activation of the invasive lab due to early cardiogenic shock. Coronary angiography revealed a critical lesion in the left circumflex artery (LCx), which was also providing collaterals to the left anterior descending artery (LAD). Percutaneous coronary intervention (PCI) was performed in the index procedure because of these findings. The patient did not have diabetes mellitus. Transthoracic echocardiography showed a left ventricular ejection fraction (LVEF) of 35% with multiple regional wall motion abnormalities.

### Diagnosis

Coronary angiogram via the right radial approach demonstrated chronic total occlusion (CTO) of the mid LAD (collateralized by left-to-left collaterals from the LCx), critical mid LCx stenosis at the bifurcation of the obtuse marginal branch (OM3), and severe proximal right coronary artery (RCA) disease (Panel A). His case was discussed with the on-call Cardiothoracic Surgery team for consideration of urgent coronary artery bypass graft, though this was deemed prohibitively high risk.

There was an inadvertent withdrawal of the guidewire after intravascular ultrasound (IVUS) imaging, but before the final angiogram was obtained. The initial PCI strategy employed a hybrid approach because the distal LCx was of much smaller caliber, whereas the proximal segment was substantially larger.

### Initial treatment

PCI to the LCx was subsequently performed with a provisional stenting technique with IVUS-guidance from the proximal to mid LCx, with a wire across OM3. Significant pinching of the OM3 was noted despite an adequate POT; hence, the struts overlying OM3 were teased open, and kissing balloon inflation and re-POT were performed, with a good angiographic result (Panel B). The procedure was performed by an interventional cardiologist with more than 10 years of experience in complex PCI and intracoronary imaging.

### Complications and their management

**Figure 1.**
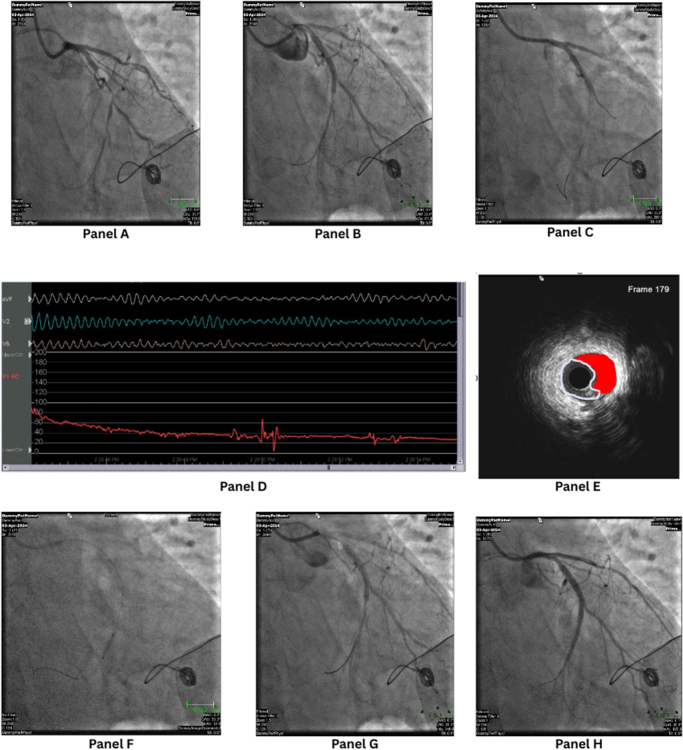
Panel A: Initial diagnostic angiogram showing critical disease of the LCx. Panel B: Angiographic results post-stenting of the LCx with optimization by kissing balloon inflation into OM3. Panel C: Wire migration and dissection with loss of distal LCx flow. Panel D: ECG and arterial trace demonstrating ventricular fibrillation (VF) and hemodynamic compromise requiring defibrillation. Panel E: IVUS image showing intramural hematoma (outlined in red) causing extrinsic compression of the true lumen. Panel F: Use of a cutting balloon to fenestrate the intramural hematoma. Panel G: Restoration of coronary flow after successful fenestration. Panel H: Final angiographic and IVUS results showing complete hematoma decompression, optimal stent expansion, and TIMI 3 distal flow. ECG, electrocardiogram; IVUS, intravascular ultrasound; LCx, left circumflex artery; OM3, third obtuse marginal branch; VF, ventricular fibrillation.

**Figure 2.**
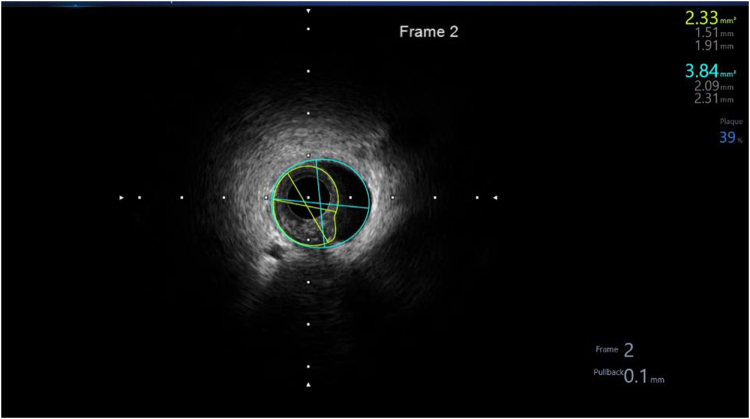
IVUS cross-sectional measurement of true lumen, false lumen, and hematoma. IVUS, intravascular ultrasound.

During the procedure, the LCx wire was seen to have migrated proximally (Panel C). An attempt to rewire distally resulted in looping of the wire and subsequent dissection and loss of flow into the distal LCx (Panel D). The patient developed VF and had to be defibrillated with subsequent ROSC (Panel E). IV amiodarone was administered (Fig. 1). The duration of ischemia before VF onset was approximately 30 seconds. Hemodynamically, just before VF, the blood pressure dropped from 110/70 mmHg to 80/50 mmHg, accompanied by sinus tachycardia at 120 bpm and marked ST-segment elevation on the arterial trace. A 150 mg IV amiodarone bolus was administered following defibrillation. IVUS imaging demonstrated an IMH with a minimal lumen area of 1.51 mm^2^, a true lumen area of 2.3 mm^2^, and a false lumen area of 1.51 mm^2^ (Fig. 2). A second wire was successfully introduced to rewire the distal LCx. IVUS confirmed true luminal position but significant intramural hematoma and collapse of the true lumen (Panel F). IVUS imaging confirmed a large IMH causing near-complete lumen compression. Following cutting balloon fenestration and stent deployment, repeat IVUS demonstrated complete hematoma decompression, adequate stent expansion, and full apposition to the vessel wall. Serial balloon dilatations with 1.25 mm and 1.5 mm compliant balloons did not restore flow. A 2.0 mm Wolverine cutting balloon was then used at the site of hematoma, inflated at 2–4 atm (Panel G). The partially inflated cutting balloon was gently rotated and advanced to create controlled micro-incisions (“fenestrations”) in the intimal layer, facilitating decompression of the IMH and restoration of flow (Panel H).

### Definitive treatment

The vessel was stented, overlapping with the initial more proximal stent. Final angiographic and IVUS images were satisfactory, with well-optimized stents and TIMI 3 distal flow (Panel I). A relook angiogram at the time of staged RCA PCI 2 days later showed widely patent LCx stents. The patient was discharged well 2 days later, with complete resolution of chest pain and marked improvement in functional status. At 1-month outpatient follow-up, the patient remained symptom-free, with no ECG changes or recurrent angina.

A summary of the clinical course and key interventions is provided in Table 1.

**Table 1:** Timeline of key events

Day/Time	Events
Day 0	STEMI presentation and urgent PCI to LCx
Day 0	Wire-induced distal dissection and VF arrest were managed successfully.
Day 2	Staged RCA PCI and relook angiogram confirming patent stents
Day 4	Hospital discharge
1-Month	Asymptomatic follow-up

LCx, left circumflex artery; PCI, percutaneous coronary intervention; RCA, right coronary artery; STEMI, ST‑elevation myocardial infarction; VF, ventricular fibrillation.

## Discussion

Iatrogenic distal coronary artery dissections resulting from guidewire manipulation are rare yet potentially fatal consequences of PCI. These occurrences may result in the development of IMH, which constricts the actual lumen, disrupts coronary blood flow, and might trigger severe outcomes such as ventricular arrhythmias or cardiac arrest^[6]^. The clinical impact depends on the extent of luminal compromise. While small, non-flow-limiting dissections may be managed conservatively, urgent intervention is warranted when there is significant flow limitation, ongoing ischemia, or hemodynamic instability.

In such cases, restoring true lumen patency can be challenging, especially when the dissection or hematoma extends distally or involves small-caliber segments. Conservative interventions, such as low-profile balloon angioplasty, are often first-line strategies aimed at decompressing the hematoma and sealing the dissection flap^[7]^. However, if these conservative techniques fail, escalation to more advanced options is necessary. Intravascular imaging, particularly IVUS, is crucial in these intricate situations. IVUS guarantees that the guidewire stays within the true lumen while offering comprehensive visualization of the hematoma’s extent, vessel dimensions, and guidewire placement – factors vital for secure and precise intervention^[8,9]^.

In this case, when compliant balloon dilatation failed to relieve the IMH, a CB was used to fenestrate the intima and decompress the IMH, restoring the lumen patency with minimal vessel trauma. Although CBs are more commonly used in resistant fibrotic or calcific lesions, emerging reports have demonstrated their utility in selected cases of SCAD with IMH^[1,10]^. By producing controlled incisions in the intimal layer, cutting balloons can create communication between the false and true lumens, allowing hematoma evacuation and restoration of vessel patency^[11]^.

While the use of CBs in SCAD has been described, their application in iatrogenic dissections, particularly in the distal coronary tree, remains underreported. Recent studies by Maričić *et al*^[1]^ highlighted that the same principle can be extrapolated to iatrogenic coronary dissections, particularly when the dissection flap and IMH exert a significant compressive effect that limits flow, and when conventional balloon angioplasty proves ineffective. In such scenarios, CB-assisted fenestration offers a mechanically precise means of addressing the underlying pathophysiology without necessitating aggressive high-pressure dilatation that might propagate the dissection or worsen vessel injury^[1]^.

In our case, the distal dissection posed an additional procedural challenge, as small-caliber vessels warranted careful handling. A 2.0 mm Wolverine CB was inflated at low pressure (2–4 atm) with controlled dottering and rotation and successfully decompressed the hematoma without causing further vessel injury. IVUS confirmed hematoma evacuation, restored lumen geometry, and optimal stent expansion. The final angiography showed full restoration of distal TIMI 3 flows with no complications, indicating the safety and effectiveness of this approach.

While the literature describing CB application in iatrogenic dissection remains sparse, this case highlights its potential as a rescue approach when conventional balloon angioplasty is ineffective due to patient comorbidities or physical constraints. Moreover, a new expert agreement recommends customized management techniques in wire-induced dissections, highlighting the relevance of intracoronary imaging and device escalation in difficult dissections with IMH^[12]^.

## Conclusions

Cutting balloons can be used at the site of a hematoma to treat IMH that occur due to inadvertent distal coronary wire dissections. The use of intracoronary imaging to ensure luminal wire position and adequately size balloons can further enhance the safety and efficacy of this technique.

## Data Availability

The data underlying this article can be shared on reasonable request to the corresponding author.

## Author notes

Both Eran Sim and Bharat Khialani were involved in the case as operators and were responsible for preparing, reviewing, and finalizing the manuscript.
